# The Phosphorus Bond, or the Phosphorus-Centered Pnictogen Bond: The Covalently Bound Phosphorus Atom in Molecular Entities and Crystals as a Pnictogen Bond Donor

**DOI:** 10.3390/molecules27051487

**Published:** 2022-02-23

**Authors:** Pradeep R. Varadwaj, Arpita Varadwaj, Helder M. Marques, Koichi Yamashita

**Affiliations:** 1 Department of Chemical System Engineering, School of Engineering, University of Tokyo 7-3-1, Tokyo 113-8656, Japan; varadwaj.arpita@gmail.com (A.V.); yamasita@chemsys.t.u-tokyo.ac.jp (K.Y.); 2 Molecular Sciences Institute, School of Chemistry, University of the Witwatersrand, Johannesburg 2050, South Africa; helder.marques@wits.ac.za

**Keywords:** pnictogen bonding, phosphorus as a pnictogen bond donor, σ- and π-hole interactions, bonding modes, sum of the van der Waals radii concept, geometry analysis, MESP analysis, IGM-*δg* analysis

## Abstract

The phosphorus bond in chemical systems, which is an inter- or intramolecular noncovalent interaction, occurs when there is evidence of a net attractive interaction between an electrophilic region associated with a covalently or coordinately bonded phosphorus atom in a molecular entity and a nucleophile in another, or the same, molecular entity. It is the second member of the family of pnictogen bonds, formed by the second member of the pnictogen family of the periodic table. In this overview, we provide the reader with a snapshot of the nature, and possible occurrences, of phosphorus-centered pnictogen bonding in illustrative chemical crystal systems drawn from the ICSD (Inorganic Crystal Structure Database) and CSD (Cambridge Structural Database) databases, some of which date back to the latter part of the last century. The illustrative systems discussed are expected to assist as a guide to researchers in rationalizing phosphorus-centered pnictogen bonding in the rational design of molecular complexes, crystals, and materials and their subsequent characterization.

## 1. Introduction

Noncovalent interactions are one of the chemical interactions (or chemical synthons) that have been explored for some time [1,2] and continue to be elucidated [3,4]. They are yet to be fully understood because their behavior is variable from system to system [5]. The variety of these interactions is a consequence of the variability of the nature of the electron density donor and acceptor participating in the molecular assembly and therefore depends on factors such as the local geometry (bond distance and intermolecular approach angle) and the electron density profile of the interacting atomic basins. Since acid–base interactions are central to chemical reactions [6], recognition processes [7,8], bond functionalization [9], catalysis [10,11,12], and self-assembly [13,14,15], a fundamental understanding and exploration of these interactions has been one of the key issues in the rapid development of research areas such as computational chemistry [16,17,18,19], crystallography [5,20], and crystal engineering [21,22].

Noncovalent interactions show up in different flavors, including, for example, hydrogen bonding [23], halogen bonding [24], tetrel bonding [25,26,27], chalcogen bonding [28], pnictogen bonding [29,30,31,32], aerogen bonding [33], van der Waals interactions [34], and several others [35]. They are the result of attractive engagements between sites of unequal charge density and are often identified to be of Coulombic origin (a positive site attracting a negative one). Anti-electrostatic noncovalent interactions have been characterized recently [36,37]. Depending on the nature of the interacting units responsible for a molecular complex system, the extent of energy contributions due to repulsion (Pauli and electrostatic, resulting from like charges) acts against orbital interactions, polarization, and dispersion as attractive forces [36]. However, the overall stability of a system is a delicate balance between all of them, in which the overall attractive contribution to the binding energy dominates.

This overview is focused on exploring the nature of the geometric modes of pnictogen bonding, manifest in some crystals and known since the latter half of the last century but explored in greater detail more recently [38,39,40,41,42,43], addressing only phosphorus-centered pnictogen bonding (or simply, phosphorus bonding). Phosphorus, P, the second element of the pnictogen family, Group 15 of the periodic table, has an electronegativity of 2.19 on the Pauling scale, which is appreciably smaller than that of N (3.04). The most commonly observed (formal) oxidation state of phosphorus in molecules and crystals is +3 and +5, although a variety of oxidation states, from −3 to 4, are known [44]. It forms compounds with coordination numbers from 1 (as in P_2_) to 9 (as in Ti_3_P) [44]. Consequently, a large number of chemical systems with various oxidation states and stereochemistry are known, and the rich chemistry of P has found a wide range of uses and applications for its compounds. Here we demonstrate and summarize the evidence that phosphorus in molecular entities, utilizing its electrophilic character, has the ability to make attractive engagements with negative sites to form crystalline materials.

*The phosphorus bond in chemical systems, which is an inter- or intramolecular noncovalent interaction, can be identified when there is evidence of a net attractive interaction between an electrophilic region associated with a covalently or coordinately bonded phosphorus atom in a molecular entity and a nucleophile in another, or the same, molecular entity. It is the second member of the family of pnictogen bonds, formed by the second member of the pnictogen family of the periodic table.*

In this overview, we reexamine several crystals deposited in the Inorganic Crystal Structure Database (ICSD) [45,46] and the Cambridge Structural Database (CSD) [47] to illustrate that many crystals have been reported in which the nature of phosphorus-centered pnictogen bonding was not always appreciated. Perhaps the underlying reason is that unlike hydrogen bonding [23], halogen bonding [24], and chalcogen bonding [28] interactions, which have been formally defined by IUPAC and have a delineated set of characteristics and signatures, pnictogen bonding has yet to be formally defined. The characteristics and features used to define the three types of noncovalent interactions mentioned above may be transferable to elements of the pnictogen family.

In this overview, we make explicit use of the “sum of the van der Waals radii” concept [48] and directional features [49,50,51] to identify pnictogen bonding in the crystals examined. In order to provide additional insight into the presence of pnictogen bonding in the examples selected, we also employ the molecular electrostatic surface potential (MESP) model [52] to examine the nucleophilic and electrophilic nature of isolated monomeric entities responsible for the stability of most of the crystals examined. In some instances, we employ the charge-density-based promolecular independent gradient model (IGM) [53,54] to provide supporting evidence of pnictogen bonding in the crystals. We stress that our aim is to bring to the reader’s attention examples of these systems, and we make no claim about the comprehensiveness of our survey.

## 2. Computational Details

As mentioned above, and in some other selected cases, the monomeric entities responsible for the formation of several crystals reviewed in the following sections were fully relaxed in the gas phase with ωB97XD/Jorge-ATZP (ATZP: an augmented triple zeta valence quality plus polarization basis set), as well as with MP2/def2-TZVPPD, or MP2/aug-cc-pVTZ. The basis sets were obtained from the basis set exchange library [55,56]. We used two different computational approaches to verify that the choice of the basis set and exchange–correlation methods do indeed provide the required insights, as an inappropriate choice of basis set can produce misleading results, especially when a trend in a specific property such as the nature of electrostatic potential on the surface of a given series of molecular entities is sought. In all cases, the second derivative of energy with respect to the atom-fixed nuclear coordinates was evaluated for the monomers. Positive eigenvalues were identified, and hence the geometries used for the evaluation of the electrostatic surface potential of monomers were all identified to be true minima.

The local most potential minima and maxima (*V_S,max_* and *V_S,min_*, respectively) on the surface of some selected monomers were calculated using the same levels of theory. The reason for evaluating these properties was to delineate the electrophilic and nucleophilic nature of various regions on the surfaces of these molecules, and hence to gain insight into why certain inter- and intra-molecular bonding interactions occur between specific atomic basins when the monomeric units are in close proximity, leading to the formation of crystalline materials. The underlying theoretical details of the MESP do not require further elaboration since these have been discussed extensively [57,58,59].

When *V_S,max_* > 0 and *V_S,min_* > 0 on the electrostatic surface of a molecular entity, this implies the presence of an electrophilic (charge-density-deficient) region. It follows that *V_S,max_* < 0 and *V_S,min_* < 0 on the surface of a molecular entity imply the presence of a nucleophilic (charge-density-rich) region [51].

When a region of an atom in a molecule, characterized by *V_S,max_* > 0 (*V_S,min_* > 0), is in close proximity with a region of an atom in a neighboring molecule that is characterized by *V_S,min_* < 0 (or *V_S,max_* < 0), it would be tempting to assume the presence of an electrostatic attraction between these two regions that drives the formation of the supermolecular structure.

The 0.001 a.u. (electrons Bohr^−3^) isoelectron density that arbitrarily defines the van der Waals surface of a molecular entity was used to compute the electrostatic potential. While any definition of a molecular surface is necessarily arbitrary [52,60], as we have demonstrated elsewhere [61,62]—and show also in this study (vide infra)—the use of the suggestions of Bader and coworkers [63], as well as others [64,65], that the 0.001/0.002 a.u. contour of charge density *ρ*(r) encompasses more than 90–95% of the molecule electronic charge is still found in many reports, especially when it concerns the electrostatic surface potential on molecular entities.

The concepts of the σ-hole [57,58,59] and the π-hole [66,67,68,69] are used throughout this article. A σ-hole is defined as a region on the surface of a covalently bonded atom, A, along the outer extension of the R–A covalent bond, that features a depletion of electron density, and R is the remaining part of the molecule [51]. A σ-hole can be positive or negative, as determined by the local most surface maximum of potential. Hence, when *V_S,max_* < 0 on the surface of atom A along the R–A bond extension, it is a negative σ-hole; if *V_S,max_* > 0, it is a positive σ-hole. Similarly, a π-hole may be defined as an electron-deficient region on the surface of a molecule, often appearing on the centroid region of arene molecules, or even on the central bonding region of a double and triple bond (for example, in the C≡C bond of acetylene), that is capable of accepting electron density from a partner molecule when it is involved in an attractive engagement with a negative site. A π-hole can be observed on the surface of nitro compounds, R–NO_2_ [70], and coordinated nitrate anions, for which an anti-electrostatic NO_3_^−^···NO_3_^−^ interaction can be anticipated [71]. Typical π-hole “hosts” containing group 13 elements can be represented by AX_3_ (A = B, Al, Ga; X = H, F, Cl [72,73,74]).

We also use concepts such as Type-I, Type-II, and Type-III topologies of bonding [51] throughout this overview to characterize the nature of pnictogen bonding in chemical systems. A Type-I topology of bonding interaction within or between a molecular entity or entities occurs when the angle θ of interaction between the pnictogen atom and the interacting site, where either both are positive or both are negative, lies between 90 and 150°. A Type-II topology has 140° < θ < 180°, yet in this case, the covalently bonded pnictogen atom features an electrophile that attracts a negative site. A Type-III topology of bonding interaction between the molecular entities occurs when the covalently bonded pnictogen atom and its interacting partner atom are both positive, or both negative, and 150° < θ < 180°.

We used the crystal geometries in some cases to evaluate the promolecular charge density based isosurfaces to confirm chemical bonding interactions, calculated within the promolecular framework of IGM-*δg* [53,54]. This approach has proven useful in providing local insight into the source of atomic domains participating in intra- and inter-molecular interactions [49,50,51]. IGM is associated with the electron density gradient, which leads to the descriptor *δg* that identifies and quantifies the ED gradient softening due to interactions. Because the total gradient of charge density |∇ρ| is attenuated to zero at the bond critical point between a pair of two atomic basins A and B, the numerator of reduced density gradient *s(r)* drops. Within the framework of IGM, promolecular atomic electron densities are summed up but the associated atomic gradients do not interfere, which is achieved by using absolute values upon summing atomic gradients, thereby erasing any electron gradient contragradience feature. This artifice brings the system into a virtual state in which individual gradients could be added, keeping only the true electron density. The resulting total gradient |∇ρ^IGM^| is then an upper limit of the true gradient, and the difference between them, *δg*, quantifies the net ED gradient collapse due to interactions. That is, the *δg* descriptor identifies the presence of opposite signs in the components of the total ED gradient |∇ρ(r)| due to interactions. As such, IGM provides an uncoupling scheme that automatically separates intra- and inter-fragment interactions in a molecular entity, which can be plotted in 2D, as well as in 3D (isosurface volumes), to reveal the presence of inter- or intramolecular interactions.

All the electronic structure calculations were performed using Gaussian 16 program package [75]. Mercury 4.0 [76], Gaussview 5.0 [77], AIMAll [78], Multiwfn [79], and VMD [80] suite of programs were utilized for analysis of, *inter alia*, the geometry, charge density topology, and electrostatic potential of the systems examined.

## 3. Illustrative Crystalline Systems

### 3.1. Polymorphs of Phosphorus

The group 15 elements have already started to play a role in the development of 2D semiconductor materials, including phosphorene, arsenene, antimonene, and nitrogene [81]. The terminology used for these monolayered materials is by analogy with graphene. Probably the most popular phosphorus-containing semiconducting systems (single-layered and bulk phosphorus allotropes) are single-layered Hittorf’s phosphorus, Hittorfene; black phosphorus, black phosphorene; and A7 phosphorene [82]. The monolayer of novel γ-phosphorus nitride (γ-PN) was shown to be a candidate for a visible-light-driven, water-splitting photocatalyst, with an indirect bandgap transition energy of 2.85 eV, based on DFT calculations [83]. Similarly, 2D black phosphorus (a puckered material) is among the family of 2D and layered materials that have distinctive crystalline symmetries and exhibit various properties, such as high carrier mobility, strong infrared responsivity, widely tunable bandgap, in-plane anisotropy, and spontaneous electric polarization [84].

**Figure 1:**
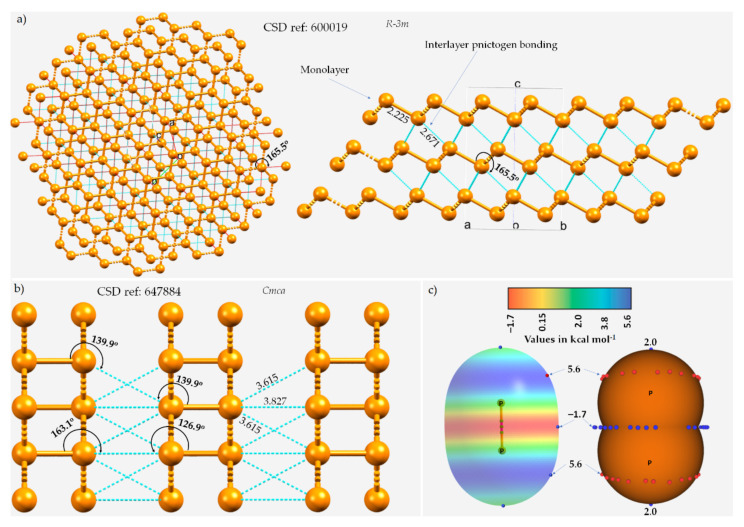
The ball-and-stick mode of the structure of (**a**) *A7* rhombohedral 0.19(P_31_)_n_ crystal of phosphorus and (**b**) orthorhombic black phosphorus, showing interlayer P···P type pnictogen bonded interactions. ICSD references and space groups for each are shown. Selected bond lengths and bond angles are in Å and degrees, respectively. (**c**) The MP2(full)/aug-cc-pVTZ level 0.001 a.u. isodensity envelope mapped electrostatic potential surface of an isolated P_2_ molecule. The tiny red and blue circles in (**c**) represent the local maxima and local minima of electrostatic potential (*V_S,max_* and *V_S,min_*, respectively). Dotted lines in cyan represent an interaction between a pair of P atoms, and those in red, as in (**a**), represent hanging contacts. The quantum theory of atoms in molecules (QTAIM)-based molecular graphs are superimposed in (**c**), and the bond paths are in atom color, accompanied by bond critical points (tiny sphere in green). The phosphorus atoms in (**a**,**b**) are colored orange.

The chemical bonding holding the monolayers together in these 2D materials is often assumed to be a consequence of van der Waals forces, which are dispersive by nature. One such instance of the layered (*A7*) rhombohedral structure of phosphorus (space group *R-3m*), in which each P site in a given monolayer is bonded to three P sites in a neighboring nearest monolayer, is illustrated in Figure 1a.

The three P···P intermolecular distances responsible for the interfacial region in the crystal are all equivalent, with *r*(P···P) = 2.671 Å and P–P···P = 165.5°, and appear slightly off the extension of the P–P covalent bonds (*r*(P–P) = 2.225 Å). In the case of black phosphorus, the P···P intermolecular distances between P sites of neighboring monolayers are 3.827 and 3.615 Å and are associated with ∠P–P···P contact angles of 163.1 and 139.9°, respectively (Figure 1b), indicative of the occurrence of Type-III and Type-I topologies of phosphorus-centered pnictogen bonding, respectively, in the crystal.

The quasilinear directional bonding features in both the crystals above may be explained using the MESP model of an isolated P_2_ molecule shown in Figure 1c. As can be seen, the surface of the P atom in P_2_ has district regions of different potential; the first is around and the second is along the P≡P bond extensions. The first region is beltlike and features several local maxima of potential (*V_S,max_* ≈ 5.6 kcal mol^−1^) that are more positive than the localized region along the P≡P bond extension (*V_S,max_* = 2.0 kcal mol^−1^). The central bonding region is equipped with a belt of negative potential, with *V_S,min_* ≈ –1.7 kcal mol^−1^. Clearly, there is no σ-hole observed on P along the P≡P bond extension (and this is also expected in the case of N_2_). There are two local minima (but not local maxima) on the outer surfaces of the two P atoms along the P≡P bond extension that are probably the result of a buildup of relatively large charge density compared to the lateral sides of the same atoms. As such, the quasilinear nature of the P···P interlayer contacts shown in Figure 1a emerges because of the attraction between a pair of P sites that feature, although both positive, dissimilar charge density. In other words, these are the result of attraction between the regions on interacting P atoms described by the local minimum and maximum of potentials, which could be regarded as lump–hole interactions [85].

**Figure 2:**
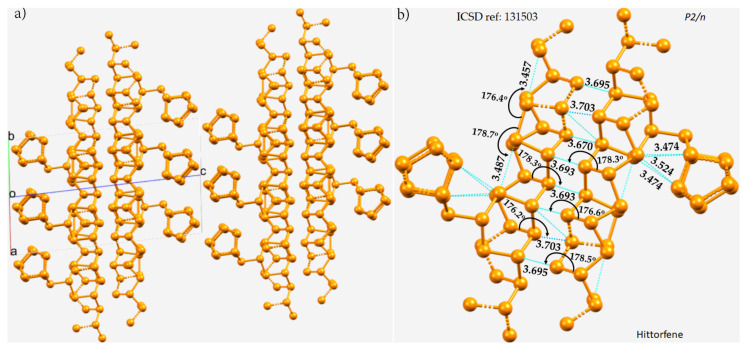
(**a**) The 2 × 2 × 2 supercell structure of Hittorfene (violet phosphorus). (**b**) The nature of P···P bonding interactions between the layers, and within the same layer, of the crystal. Selected bond distances and bond angles are in Å and degrees, respectively. Dotted lines in cyan represent an interaction between a pair of P atoms. P atoms in the ball-and-stick models are colored orange.

The crystal structure of violet phosphorus (Hittorfene) has been reported recently [86]. This layered, 2D semiconducting phosphorus allotrope (monoclinic space group) has an optical band gap of 1.7 eV. It undergoes thermal deposition at 52 °C, which is higher than the decomposition temperature of black phosphorus. The nature of P···P bonding in Hittorfene is very different from that observed in black phosphorus. One of the crucial features of Hittorfene is that each layer is bonded to the nearest layer by a number of P···P contacts with a variety of bond distances between them (Figure 2a). The contacts are strongly directional (∠P–P···P between 176 and 179°). There are also intralayer P···P contacts; they are markedly shorter than the interlayer contacts and are directional. They are all less than twice the vdW radius of P, 3.80 Å, and hence can be regarded as phosphorus bonds.

There are also a large number of phosphorus-containing crystal structures deposited in the CSD and ICSD databases. Many have been known for some considerable time, including, for example, (P_2_)*_n_*, ICSD ref. 647884 [87]; PCl_4_, ICSD ref. 26594 [88]; PCl_5_, ICSD ref. 26661 [89] and ICSD ref. 29124 [90]; PBr_5_, ICSD ref. 15559 [91]; P_4_S_3_, ICSD ref. 16711 [92]; P_4_S_5_, ICSD ref. 16681 [93]; P_4_S_7_, ICSD ref. 23842, and P_4_S_10_, ICSD ref. 174008 [94]; I_2_P_4_S_3_, ICSD ref. 26485 [95]; and P(CN)_3_, ICSD ref. 16587 [96]. In these systems, the covalently bonded phosphorus in a given molecular entity interacts attractively with the Lewis base in partner molecule(s), thus forming phosphorus-centered pnictogen bonding and contributing to the structure of the crystals.

What follows is a description of a number of crystal systems wherein chemical bonding involving phosphorus bonds plays a structure-determining role. For many of them, we examined their MESP to provide insight into their Lewis acid behavior necessary for the formation of an acid–base interaction. Isolated chemical systems could serve as examples of phosphorus bonding. Chandra and coworkers [97], and others [98], have reported such chemical systems expounded from studies of isolated entities. They showed that phosphorus can serve as an acceptor of electron density from the π electron cloud in partner molecules. In particular, they have shown that the P···π phosphorus bonding interactions are responsible for the stability of the PCl_3_–C_2_H_2_ and PCl_3_–C_2_H_4_ heterodimers and similar trimers and tetramers generated during low-temperature measurements. The dominance of phosphorus bonding in the PCl_3_–C_2_H_2_ and PCl_3_–C_2_H_4_ heterodimers over other interactions (such as H···π, H···Cl, H···P, Cl···π, and lone pair–π interactions) was also discussed.

### 3.2. Phosphorus Trihalides

**Figure 3:**
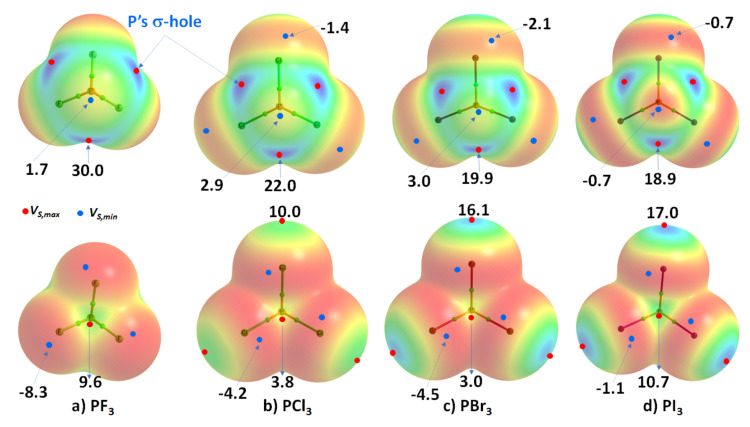
Comparison of ωB97XD/Jorge-ATZP calculated 0.001 a.u. isodensity envelope mapped potential on the electrostatic surfaces of PX_3_ (X = (**a**) F, (**b**) Cl, (**c**) Br, (**d**) I) molecules. Selected tiny circles in red and blue describing *V_S,max_* and *V_S,min_* values in kcal mol^−1^ are shown, which are the local maxima and minima of potential, respectively. Two views of each MESP graph are displayed for each molecule. Top: coordinated P faces the reader. Bottom: the three X atoms forming a triangular architecture face the reader. The QTAIM-based molecular graphs are superimposed on each case, and the bond paths are in atom color, accompanied by bond critical points (tiny sphere in green).

Phosphorus trihalides, PX_3_ (X = F, Cl, Br, I), are probably the simplest tetra-atomic molecular systems that can be used to arrive at an understanding of phosphorus-centered pnictogen bonding in crystals. The ωB97XD/Jorge-ATZP computed MESP plots for PX_3_ (X = F, Cl, Br, I) are compared in Figure 3. Except for F in PF_3_ (Figure 3a), the halogen derivative X has a charge density depletion region on the outer extensions of the P–X covalent bond that increases as the electronegativity decreases in the series from Cl through Br to I. Associated with these depleted charge density regions are σ-holes on the halogen derivatives, whose strength increases in the opposite order: P–Cl (10.0 kcal mol^−1^) < P–Br (16.1 kcal mol^−1^) < P–I (17.0 kcal mol^−1^), concordant with an increase in the polarizability of X. The apparent absence of a σ-hole on F in PF_3_ is not unexpected and has been observed in other chloro- and fluorinated compounds [60,61,62,99].

On the other hand, the strength of the σ-hole on P decreases as X proceeds from F down to I and so is most stable along the outermost X–P bond extensions when X = F. This is expected given that F is the most electronegative and least polarizable of the halogens. It therefore has a very high ability to pull electron density on P towards the bonding region in the P–F bonds, thus leaving a strongly positive potential on the surface of covalently bonded P.

We observe that the σ-hole regions on covalently bonded X are more positive than the lateral portions in PX_3_. The latter is described by a belt of negative potential. Except for PF_3_, the belt is not equipotential as can be inferred from the color of the belt. For example, the potential around the lateral site shown in the top view of Figure 3b–d is colored orange (*V_S,min_* roughly −1.4 kcal mol^−1^ for PCl_3_), and that shown in the bottom view is colored red (*V_S,min_* ~ −8.3 kcal mol^−1^ for PCl_3_). The P site in PX_3_ (X = F, Cl, Br, with I being the exception) is entirely positive (i.e., both *V_S,max_* > 0 and *V_S,min_* > 0). These results suggest that the lateral and axial sides, respectively, of halogen derivatives in a given PX_3_ molecule might be capable of making an attractive engagement with the axial and lateral sites, respectively, of another molecule of the same type to form X_3_P···PX_3_ complexes, as we point out below.

While the (arbitrary) use of the 0.001 a.u. isodensity envelope is often recommended for computing electrostatic potential [100,101], it may not always be appropriate [60,61,62]. Its use in the present case, for instance, suggested that *V_S,max_* is neutral on the extensions of the three P–F bonds in PF_3_. Moreover, it is expected that the region dominated by the lone pairs on F, located around the lateral sites of the P–F bond, should be accompanied by at least three critical points of potential, *V_S,min_*. However, our calculation gave a single local minimum of potential on each F, with *V_S,min_* of –8.3 kcal mol^−1^, and a positive minimum of *V_S,min_* of +1.8 kcal mol^−1^ on P. Our calculation also gave three, nearly equivalent *V_S,max_* on P of 30.0 kcal mol^−1^ on P along F–P bond extensions, and one on the central region formed by the triangular face formed by three F atoms, with *V_S,max_* of +9.3 kcal mol^−1^. Although most of the positive and negative regions on the surface of the PF_3_ originated from a mapping of the 0.001 a.u. isoelectron density envelope, it failed to provide any insight as to whether a covalently bonded F atom in the molecule has a σ-hole on the P–F bond extensions.

**Figure 4:**
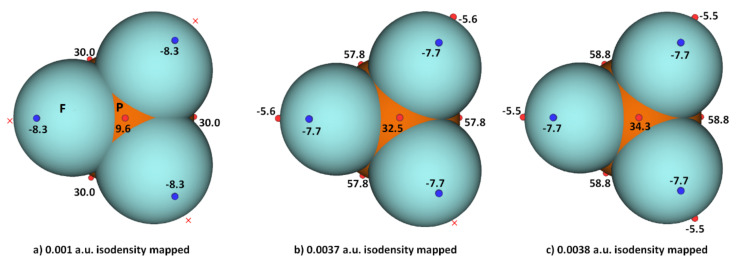
Comparison of ωB97XD/Jorge-ATZP level computed local maxima and minima of electrostatic potential of PF_3_ mapped on three different isoelectron density surfaces ((**a**) 0.001 a.u.; (**b**) 0.0037 a.u.; (**c**) 0.0038 a.u.), showing the dependence of *V_S,max_* and *V_S,min_* on the value of isodensity envelope used. The red crosses indicate the missing maxima on covalently bonded F. The tiny circles in red and blue represent the *V_S,max_* and *V_S,min_*, respectively. Values in kcal mol^−1^.

We explored the MESP of PF_3_ generated with different isodensity envelopes. These led to the emergence of the expected potential profile on the axial portion of P–F bond extensions. Specifically, two (three) local maxima of potential appeared only when we used a 0.0037 a.u. (0.0038 a.u.) isoelectron density envelope (*V_S,max_* = −5.5 kcal mol^−1^), showing that F in PF_3_ does indeed have a negative σ-hole. The values of *V_S,max_* and *V_S,min_* calculated using three different isoelectron density envelopes are shown in Figure 4. The actual nature of the potential on the molecular electrostatic surface of the PF_3_ was obtained only when the 0.0038 a.u. isodensity envelope was used. Because *V_S,max_* and *V_S,min_* on F along and around the P–F bond extensions are both negative regardless of the isodensity envelops used, it is clear that F can act as a versatile pnictogen bond acceptor.

**Figure 5:**
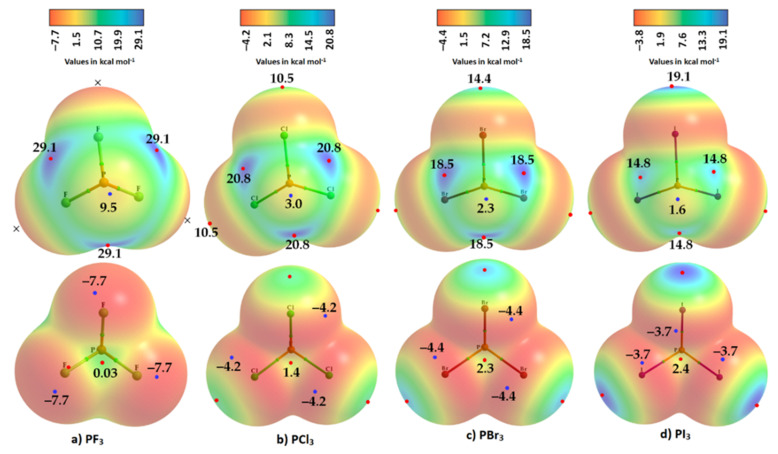
Comparison of MP2(full)/def2-TZVPD calculated 0.001 a.u. isodensity envelope mapped potential on the electrostatic surfaces of PX_3_ (X = (**a**) F, (**b**) Cl, (**c**) Br, (**d**) I) molecules. Selected *V_S,max_* and *V_S,min_* values in kcal mol^−1^ are shown, which are the local maxima and minima of potential, respectively. Two views of each MESP graph for each molecule are displayed: (Top) Covalently bonded P faces the reader. (Bottom) The three X atoms forming a triangular architecture face the reader. The QTAIM-based molecular graphs are superimposed on each case, and the bond paths are in atom color, accompanied by bond critical points (tiny sphere in green).

Because of the inconsistencies above, we recalculated the electrostatic potential using the def2-TZVPD basis set, in conjunction with MP2(full). All the monomers were optimized at this level of theory, and the wavefunctions were then evaluated at the same level. The results are summarized in Figure 5 and Table 1. The data show that the 0.001 a.u. isodensity envelope is not a suitable choice on which to compute the potential since it is not ideal to elucidate the van der Waals surface of the PF_3_ molecule. When at least a 0.0028 a.u. isodensity envelope was used for computing the potential, all the expected critical points of the potential showed up on the surfaces of the three F atoms of the PF_3_ molecule along the P–F bond extensions, as observed on surfaces of X of the other three PX_3_ molecules (Figure 5). All three σ-holes on the three F atoms in PF_3_ are found to be entirely negative (*V_S,max_* = –5.3 kcal mol^−1^), surrounded by a belt of negative sites around their lateral portions.

The σ-holes on X in PX_3_ (X = Cl, Br, I) are found to be positive, increasing in magnitude with the polarizability of X: Cl (10.5 kcal mol^−1^) < Br (14.4 kcal mol^−1^) < I (19.1 kcal mol^−1^). This is opposite to the trend found for the π-hole on the P atom in these molecules, as well as of the σ-hole on the P atom along the X–P bond extensions—a trend which is very similar to that of the *V_S,min_* around the halogen atoms in the series (PF_3_ > PCl_3_ > PBr_3_ > PI_3_). Totals of 10 and 14 local minima, respectively, were found on the surfaces of PCl_3_ and PBr_3_; these minima were significantly larger than those on PF_3_ and PI_3_ (not shown). These results show that the choice of an appropriate basis set and an isodensity envelope is important in determining the correct nature of local surface extrema and hence in being able to deduce the reactivity of a specific atomic domain in a molecular entity.

A crystalline structure of PF_3_ is unavailable; its molecular structure has been reported by gas-phase electron diffraction [102]. Crystalline PCl_3_ has been reported (at −110 °C, ICSD ref. 32027 [103], and at −150 °C, ICSD ref. 27798 [104]; Z = 4; space group *Pnma*), as has PBr_3_ (ICSD ref. 8052, Z = 2, space group *Pnma* [105]) and PI_3_ (ICSD ref. 311, Z = 4, space group *P6_3_* [106]).

**Figure 6:**
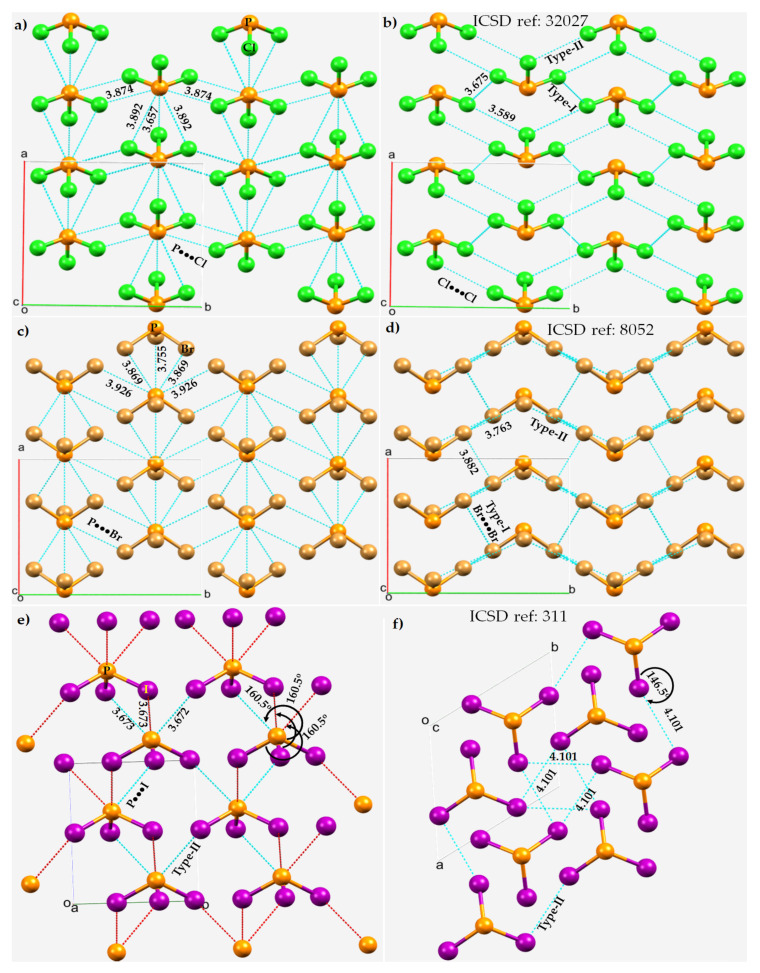
P···X and X···X bonding modes in the 2 × 2 × 2 supercell crystal structure of PX_3_ (X = Cl, Br, I). Selected bond lengths and bond angles are in Å and degrees, respectively. Atom type is shown in each case. (**a**) P···Cl and (**b**) Cl···Cl in PCl_3_. (**c**) P···Br and (**d**) Br···Br in in PBr_3_. (**e**) P···I and (**f**) I···I in PI_3_.

In a simple Lewis structure of each of these molecules, trivalent P is σ-bonded with three halogens in a triangular pyramidal structure, with a lone pair on the phosphorus atom and with three lone pairs on each halogen. The crystal structures of PX_3_ (X = Cl, Br, I) are shown in Figure 6. In each case, the P···X and X···X intermolecular bonding modes are illustrated. The average P···X bond distance is longer than the average X···X bond distance in PCl_3_ and PBr_3_. Each P site in these two structures is involved in five P···X contacts with the surrounding molecules, showing a similar pattern of intermolecular pnictogen bonding environment in both the crystals. Two of these contacts are quasilinear and the other three are nonlinear. For instance, a pair of two quasilinear Type-II interactions in PCl_3_ have ∠P–Cl···Cl = 175.3°, and the nonlinear Type-II interactions have ∠P–Cl···Cl = 143.2°. The corresponding values in PBr_3_ are 171.4 and 122.9°, respectively.

The short Cl···P contact in PCl_3_ at 3.657 Å and P–Cl···P of 171.4° (Figure 6a) may not be a pnictogen bond since the positive potential on P (*V_S,min_* = 3.0 kcal mol^−1^) in one molecule is weaker than that of the interacting Cl atom in the partner molecule along the P–Cl bond extension (*V_S,max_* = 10.5 kcal mol^−1^). This interaction is more likely a Type-III halogen bond [51], which occurs between interacting sites that feature a very similar directional behavior to a Type-II interaction, but are formed between sites with potentials of identical sign. The short P···Br contact in PBr_3_ at 3.755 Å with ∠P–Br···P = 126.8° between positive P and negative Br (Figure 6c) is nonlinear and is probably a π-centered pnictogen bonding interaction. We note further that each PBr_3_ molecular unit in the crystal is also bonded to another PBr_3_ (back-to-back in Figure 6c, but not visible) via a Br···P contact, with a bond distance and ∠P–Br···P of 4.329 Å and 158.7°, respectively (not shown). Its characteristic is similar to those of the corresponding Cl···P interactions observed in the PCl_3_ crystal, and hence we attribute this to a Type-II halogen bond.

Although the Type-II Br···Br noncovalent links force the PBr_3_ molecules to produce a zig-zag pattern along the crystallographic *b*-direction, they are weak and possibly dispersion-driven since the intermolecular distances associated with these interactions (*r*(Br···Br) = 3.763 Å) are slightly larger than twice the vdW radius of Br, 3.72 Å. This does not necessarily mean that the contribution due to electrostatics is negligible; the overall stability of these weak interactions may be understood as a delicate balance between attractive and repulsive forces. In any case, each covalently bonded Br is involved in forming at least four Br···Br contacts. Three of them are Type-I, with *r*(Br···Br) 3.766 or 3.882 Å. These are also expected to be weaker than, or of comparable strength to, the Type-II Br···Br contacts in PBr_3_. A similar conclusion may be drawn in the case of the PCl_3_ system when the P···Cl and Cl···Cl contacts are compared.

The nature of the noncovalent bonding deduced for PCl_3_ and PBr_3_ is different in crystalline PI_3_, as can be inferred by comparing the Type-II P···I and I···I bond distances shown in Figure 6e,f, respectively. For the latter system, the P···I contacts appear to be more Coulombic in nature and the I···I contacts are dispersion-driven and weak, since *r*(P···I = 3.673 Å) is significantly shorter than the sum of the vdW radii of P and I, 3.94 Å. Similarly, the I···I intermolecular distances, *r*(I···I) = 4.101 Å, are slightly larger than twice the vdW radius of I, 4.08 Å, and are nonlinear (∠P–I···I =146.5°).

It is evident that the Type-II P···X and X···X pnictogen bonding and halogen bonding modes interplay to stabilize the crystals; they can be explained by means of the MESP models of isolated PX_3_ molecules (see above). However, this model fails to explain the Type-I X···X interactions regardless of the chemical system investigated since these interactions generally occur between negative lateral sites on a covalently bonded halogen derivative that attract each other when interacting molecular entities are in close proximity.

### 3.3. Phosphorus Tricyanide

**Figure 7:**
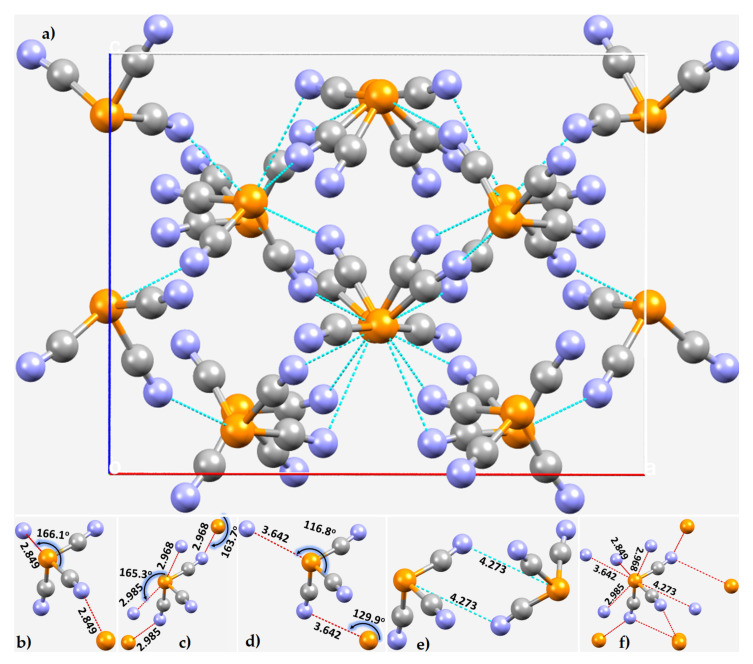
(**a**) The crystal structure of phosphorus tricyanide, P(CN)_3_, (ICSD ref. code 16587). (**b**–**f**) The nature of P···N intermolecular bonding modes between the P(CN)_3_ units in the crystal. Selected bond lengths and angles in Å and degrees, respectively. Dotted lines in red/cyan between N and P atoms (balls colored blue and orange, respectively) represent pnictogen bonding.

Emerson and Britton reported the crystal structure of phosphorus tricyanide, P(CN)_3_, in 1964 [107]. The unit cell of the crystal, containing 16 molecules of P(CN)_3_, is shown in Figure 7a.

Pyramidal P(CN)_3_ forms trigonal crystals. The local bonding topology around P and the covalently bonded N-site is depicted in Figure 7b–f. Our analysis indicates that P in each molecular entity is tetrafurcated, donating four pnictogen bonds. The closest intermolecular interaction, at a distance of 2.849 Å, occurs between the lone pairs of two N-sites in a given P(CN)_3_ unit and the P atom of two adjacent molecules (∠C–P···N = 166.1°, Figure 7b). Two pairs of P···N bonds are present at distances of 2.968 and 2.985 Å, with corresponding bond angles of 163.7 and 165.3° (Figure 7c). There is a pair of intermolecular P···N contacts at a bond distance of 3.642 Å (Figure 7d), with different angles of approach, ∠C–P···N = 116.8 and 129.9° (Figure 7d). Another pair of contacts between two units of P(CN)_3_ was found at 4.273 Å. They are formed between the lone-pair density on N in a P(CN)_3_ and the center of the triangular face (π-hole) on P of a neighboring P(CN)_3_ (Figure 7e).

Because CN has π-density around its triple bond, it is expected that π*···*π interactions between the CN moieties play an important role in the local stability of the P···N interactions. In essence, our analysis suggests that tricyanide coordinated P can act as a tetrafurcated pnictogen bond donor, whereas N serves as a bifurcated electron-density acceptor (Figure 7f).

**Figure 8:**
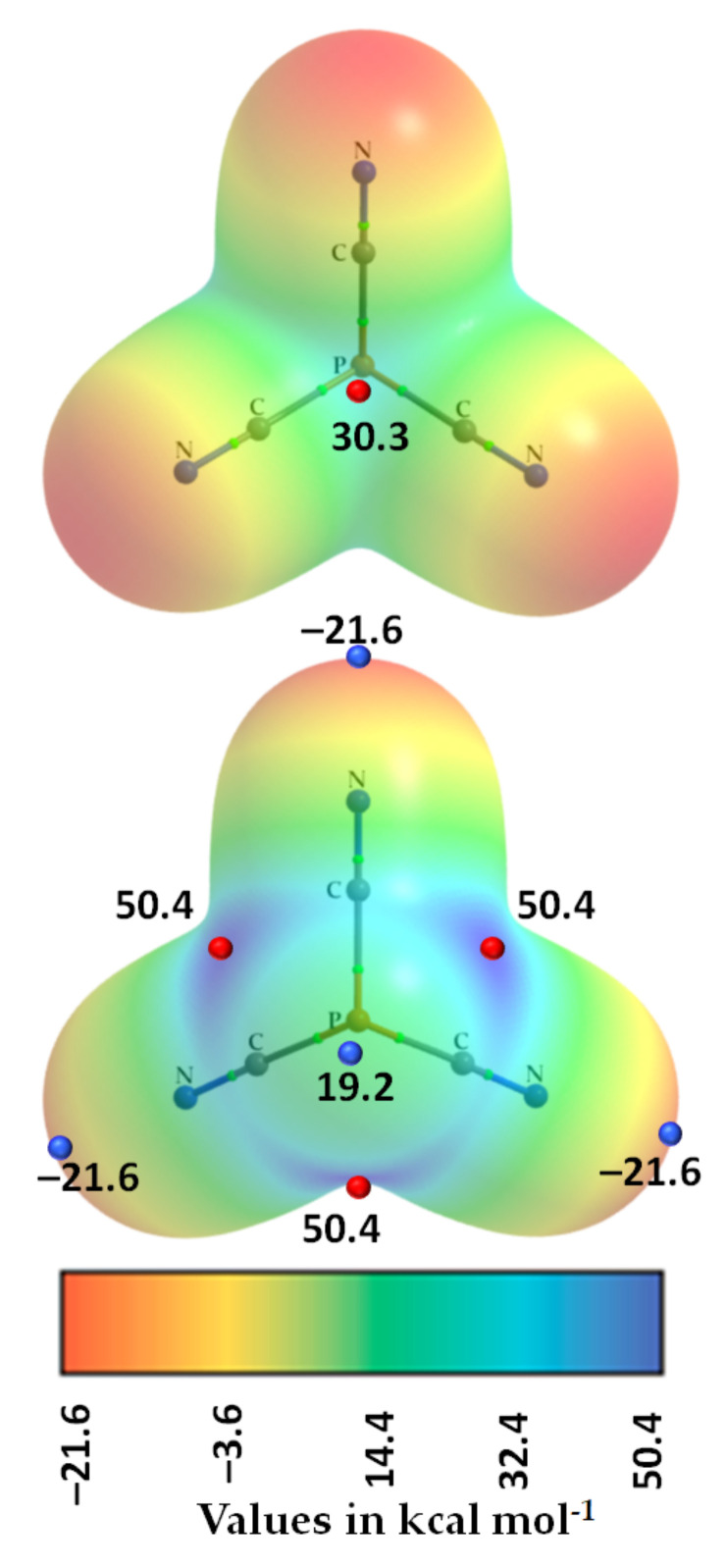
MP2(full)/def2-TZVPPD level molecular electrostatic surface potential plot of an isolated P(CN)_3_ molecule. The geometry of the molecule was optimized at the same level of theory. The 0.001 a.u. isodensity envelope was used to compute the electrostatic potential. The tiny spheres in red and blue represent *V_S,max_* and *V_S,min_*, respectively. The QTAIM-based molecular graphs are superimposed on each case, and the bond paths are in atom color, accompanied by bond critical points (tiny sphere in green). The atom type is shown.

The formation of these P···N interactions is concordant with the MESP plot shown in Figure 8 for an isolated P(CN)_3_ molecule. Covalently bonded P has three strong, equivalent σ-holes along the outer extensions of three C–P bonds (*V_S,max_* = 50.4 kcal mol^−1^). The outer surface of N along and around the C≡N bond extension presents a negative electrostatic potential (*V_S,min_* = –21.6 kcal mol^−1^). The π-density on the surface of the P atom along the outer extensions of the *C_3_* axis is also strongly positive and hence has the ability to accept electron density from negative sites. Thus, the appearance of P(π)···N and P(π)···N interactions noted above corresponding to intermolecular bond distances of 3.642 and 4.273 Å, respectively, are not surprising since they are of Coulombic origin. The detailed topology of intermolecular pnictogen bonding interactions formed by a molecular unit of P(CN)_3_ is shown in Figure 7f.

### 3.4. Phosphoryl Halides

The molecular structures of phosphoryl halides, POX_3_ (X = F, Cl, Br, I), and thiophosphoryl halides, PSX_3_, have been known for some time [108], and experimental and structural details for many of them can be found on the NIST database [109]. Their reaction with a variety of reagents can lead to the formation of solid products. For instance, the reaction of POF_3_ with dimethylamine produces solid dimethylaminophosphoryldifluoride, (CH_3_)_2_NP(O)F_2_ [110].

The ICSD contains the structures of POF_3_ (ICSD 250498) [111], POCl_3_ (ICSD 9128) [112], and POBr_3_ (ICSD 9137 [113] and 23243 [114]), but not of PIO_3_. What follows is a description of the MESP plots for isolated POX_3_ molecules (X = F, Cl, Br, I), followed by a discussion of the nature of intermolecular interactions found in the solid-state materials.

**Figure 9:**
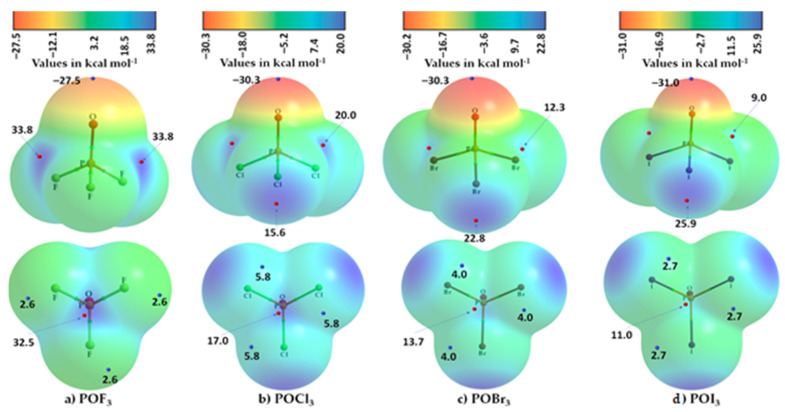
MP2(full)/def2-TZVPPD level molecular electrostatic surface potential plot of POX_3_ (X = (**a**) F, (**b**) Cl, (**c**) Br, (**d**) I) molecules. The geometry of each molecule was optimized at the same level of theory. The 0.001 a.u. isodensity envelope was used to compute the electrostatic potential. The tiny circles in red and blue represent *V_S,max_* and *V_S,min_*, respectively, with values in kcal mol^−1^. The QTAIM-based molecular graphs are superimposed for each case, and the bond paths are in atom color, accompanied by bond critical points (tiny spheres in green between bonded atomic basins). The atom type is shown. (Top) The halogen atom in POX_3_ faces the reader. (Bottom) The phosphorus atom in POX_3_ faces the reader.

The MESP plots for the four members of the POX_3_ family are shown in Figure 9. In all four cases, the axial and lateral portions of covalently bonded O are entirely negative. The *V_S,min_* along the P=O bond extension is found to be smallest in POF_3_, –27.5 kcal mol^−1^, and increases monotonically across the series, with decreasing electronegativity of X, with the largest value, at –31.0 kcal mol^−1^, found for O in POI_3_. The basicity of the Lewis base, O, is therefore largest in POI_3_. Clearly, the capacity of X to polarize the electron density of O towards the P=O bonding region in POX_3_ decreases with the electronegativity of X. This trend in potential is opposite to that observed on the surface of P along the O=P bond extension. The trend in the σ-hole’s potential on P follows the order (Figure 9) POF_3_ (32.5 kcal mol^−1^) < POCl_3_ (17.0 kcal mol^−1^) < POBr_3_ (13.7 kcal mol^−1^) < POI_3_ (11.0 kcal mol^−1^), and the Lewis acidity of P decreases with a decrease in the electronegativity of X.

It is clear (Figure 9) that P in each POX_3_ molecule has four σ-holes. Three of them are along the X–P bond extensions and are equivalent. The fourth one is along the O=P bond extension (*vide supra*). The strength of the σ-holes on P along the X–P bond extension follows the order POF_3_ (33.8 kcal mol^−1^) > POCl_3_ (20.8 kcal mol^−1^) > POBr_3_ (12.3 kcal mol^−1^) > POI_3_ (9.0 kcal mol^−1^). Therefore, the P atom in POX_3_ may interact productively with a negative site in another molecule when they are in close proximity.

X in each POX_3_ was found to be positive along the P–X bond extensions (except for the P–F bond extensions in POF_3_). We observed a systematic increase in the potential associated with the σ-holes on X in POX_3_ (X = Cl, Br, I) because of the increasing polarizability of X from Cl through to I, and hence an increase in the stability of the σ-holes in the order P–Cl < P–Br < P–I. Although the halogen derivative in these POX_3_ entities is entirely positive, the axial site is more positive than the lateral portions.

By contrast, the F atoms along the P–F bond extensions in POF_3_ are found to be neutral with no local maxima found on their surfaces. This conclusion was arrived at using the potential evaluated on the 0.001 a.u. isodensity envelope. Our further calculation using the 0.0022 a.u. isodensity envelope indicated that the σ-holes on the F atoms along the P–F bond extensions in POF_3_ cannot be neutral. The electron density on the lateral and axial portions is not the same; it is inherently anisotropic, with *V_S,min_* and *V_S,max_* of 4.0 and 6.8 kcal mol^−1^, respectively. This apparent inconsistency, as we have already remarked (*vide supra*), is the result of the arbitrary use of the 0.001 a.u. isodensity envelope to compute the potential. Similar potentially misleading conclusions have been discussed previously about different chemical systems [60,61,62], and it was demonstrated that an appropriate choice of the isodensity envelope could assist in extracting the actual nature of the potential that might explain why certain sites on the surfaces of some atoms in some molecules (e.g., CH_3_Cl [62,115] and CF_4_ [99]) attract other atoms in another molecule to form molecular complexes and crystals [51,60,116].

**Figure 10:**
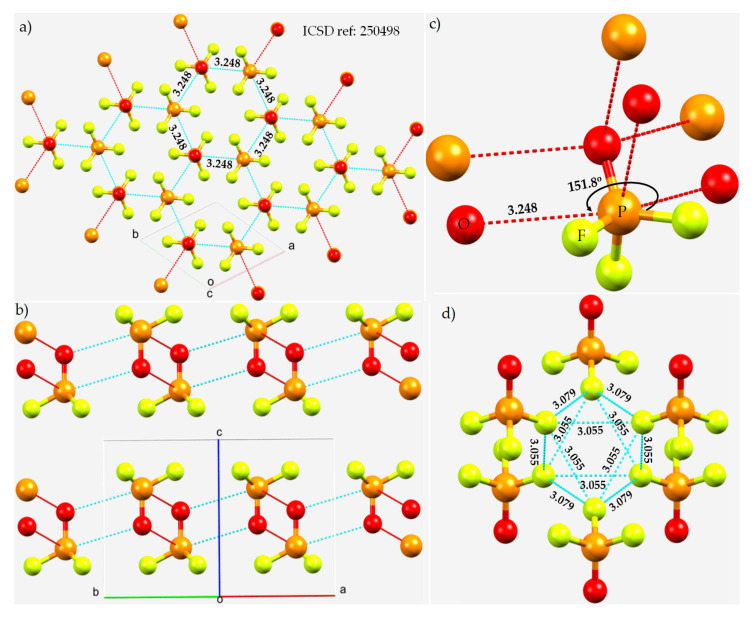
(**a**,**b**) Illustration of two different views of phosphorus bonding in the 3 × 3 × 3 and 2 × 2 × 2 supercell structures of the crystal of POF_3_ (space group: *P*$\bar{3}$*m1*), with the former showing a hexagonal pattern of bonding within a given layer and the latter showing a layer-like structure. (**c**) The local pattern of P···O bonding around each POF_3_ molecular entity in the crystal. (**d**) The local pattern of F···F bonding between the POF_3_ molecules in the crystal. Atom type is shown in (**c**), and selected bond distances and bond angles are given in Å and degrees, respectively.

The dominant attraction between the POF_3_ molecules in crystalline phosphoryl fluoride crystal (trigonal space group *P*$\bar{3}$*m*1) [111] (Figure 10a) arises from the electrostatic interaction between the sites of opposite potential localized on the surfaces of O and P, thus forming F–P···O pnictogen bonds along the F–P bond extensions. Each O accepts three pnictogen bonds, and P in the same molecule donates three pnictogen bonds (Figure 10b). They are all equivalent and directional, with *r*(P···O) and ∠F–P···O values of 3.248 Å and 151.8°, respectively. They are all less than the sum of the vdW radii of O and P, 3.4 Å. They form a zig-zag, chainlike pattern of molecular architecture (Figure 10c). The Raman spectra of neat and matrix isolated POF_3_ display an extra line that signified the presence of intermolecular interaction in the solid state [111].

In crystalline POF_3_, the F atoms in a POF_3_ molecule are in attractive engagement with the F atoms of a neighboring POF_3_ molecule (Figure 10d). Each of them forms at least four F···F contacts, displaying the versatility of F to serve both as an acceptor and a donor of electron density. When it donates Type-II σ-hole bonds, the bond distance, *r*(F···F), and the bond angle, ∠P–F···F, are 3.079 Å and 140.9°, respectively. When F is involved in Type-I contacts, ∠P–F···F = 128.5° and *r*(F···F) = 3.055 Å. The intermolecular F···F distances are all slightly greater than twice the vdW radius of F, 2.92 Å, suggesting that these are dispersion-driven F···F interactions [60,61,116,117,118]. The detailed geometrical topology of Type-I and Type-II F···F interactions is shown in Figure 10d. The stability of the POF_3_ crystal is also partly driven by F···O halogen bonding interactions in the crystal (not shown). All the Type-II intermolecular bonding interactions are consistent with what can be expected of a Coulombic interpretation that emerged from the MESP model of POF_3_.

**Figure 11:**
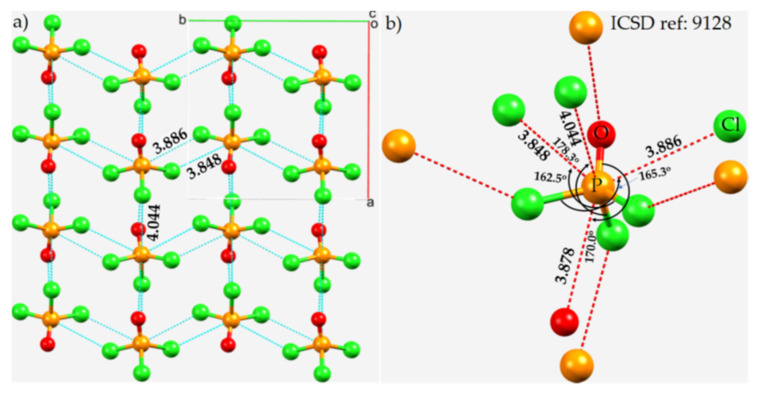
(**a**) The 2 × 2 × 2 supercell structure of the crystal of POCl_3_ (space group: *Pn2_1_a*), showing phosphorus-centered Type-II P···Cl pnictogen bonds. (**b**) The local topology of Type-II P···Cl and P···O pnictogen bonds formed along the extensions of the Cl–P and O=P covalent bonds in each POCl_3_ molecule, respectively. Type-I and -II Cl···Cl contacts between the POCl_3_ molecules in the crystal are not shown. Selected bond distances and angles are in Å and degrees, respectively. Atom type is shown in (**b**). Dotted lines between molecular entities in cyan represent intermolecular interactions, and the hanging contacts are represented by dotted lines in red.

P···O intermolecular interactions are a notable feature in crystalline POCl_3_ (Figure 11a) [112]. However, in this case, the positive center P is a donor of four nonequivalent pnictogen bonds (Figure 11b). Three of them are along the extension of three Cl–P covalent bonds; one is a P···O interaction (*r*(P···O) = 3.878 Å) and two are P···Cl interactions (*r*(P···Cl) = 3.848 and 3.886 Å). The P···O bond distance is larger than the sum of the vdW radii of P and O (3.4 Å), and both P···Cl bond distances are larger than the sum of the vdW radii of P and Cl (3.72 Å). They are all directional, yet quasilinear. The longer pnictogen bond has *r*(P···Cl) = 4.044 Å and ∠Cl–P···Cl = 162.5°, and it is formed between the P(π) density localized on along the *C_3_* bond axis in one molecule and the lateral portion of the Cl atom in a neighboring POCl_3_ molecule (Figure 11b). In addition, the crystal also features several Type-I and Type-II halogen bonded contacts, among others (not shown).

**Figure 12:**
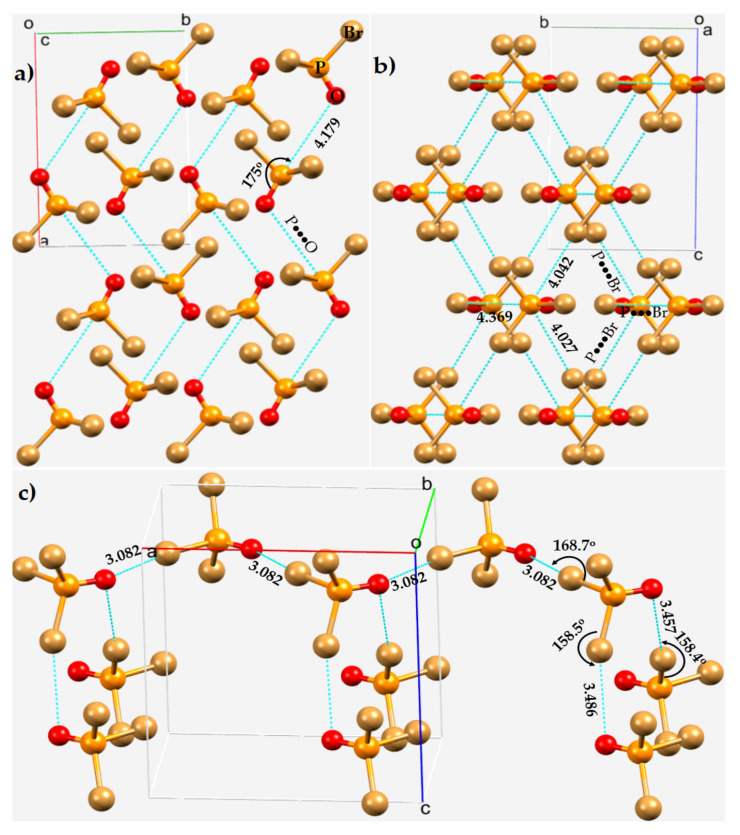
Ball-and-stick model of the crystal structure of POBr_3_ (ICSD ref code: 23243), showing the simplified network of (**a**) P···O and (**b**) P···Br noncovalent interactions between POBr_3_ units. (**c**) The pattern of Br···O halogen bond in the crystal. Dotted lines in cyan between atoms represent intermolecular interactions. Selected bond distances and bond angles are in Å and degrees, respectively. Atom type is shown in (**a**).

Phosphoryl bromide, POBr_3_, crystallizes in the orthorhombic space groups *Pn2_1_a* and *Pnma* [112,114]. Figure 12a,b displays the 2 × 2 × 2 supercell structure of the *Pn2_1_a* form [114]. The unit cell contains four molecules of POBr_3_. As found with POF_3_ and POCl_3_, the phosphorus atom is pentavalent. The shortest intermolecular distance between the Br and O is 3.082 Å, with ∠P–Br···O = 168.7°. There are two such interactions formed by a single molecule of POBr_3_ upon interaction with two same neighboring molecules, resulting in a zig-zag chain along the crystallographic *a*-direction (Figure 12c). There are two contacts formed between the nucleophilic sites on O in one molecule of POBr_3_ Br on two neighboring molecules. The intermolecular distances associated with these two contacts were 3.486 and 3.457 Å, with ∠P–Br···O of 158.5 and 158.4°, respectively (Figure 12c). Other than these prominent directional bonding features, the crystal also features Br···Br Type-I and Type-II halogen bonded contacts (not shown).

**Figure 13:**
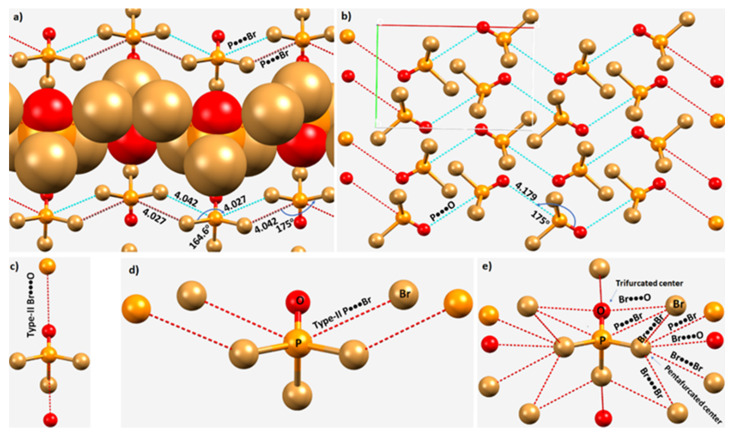
(**a**,**b**) The zig-zag nature of the P···Br and P···O interactions, respectively, in POBr. (**c**,**d**) Schematic representation of Type-II Br···O and P···Br interactions, respectively. (**e**) A tentative representation of the intermolecular interactions formed by each POBr_3_ unit with the neighboring molecules in the crystal (ICSD ref. code 23243), marked by hanging dotted lines in red between atoms. Selected bond distances and bond angles are in Å and degrees, respectively.

Each P in POBr_3_ is engaged in four long-ranged contacts with the negative site of surrounding molecules, along the extension of the Br–P and O=P bonds. Three of them are P···Br interactions and the other is a P···O interaction, an intermolecular bonding topology which is very similar to that in crystalline POCl_3_ (vide supra). The contacts are all of unequal length; the P···Br distances are longer than the P···O bond distance (Figure 12a,b). Both the P···Br and P···O contacts are responsible for the zig-zag chainlike molecular architectures as shown in Figure 13a,b, respectively. The intermolecular distance and angle of interaction associated with the Br–P···O contacts are 4.179 Å and 175.0°, respectively (Figure 12a). They exhibit strong directional feature competitive with Type-II P–Br···O halogen bonds. The intermolecular distance for Br···O halogen bonds is less than the vdW radii sum of Br and O atoms, 3.36 Å, while the P···O pnictogen bonds are significantly larger than the vdW radii sum of P and O atoms, 3.40 Å. These contacts are all characteristics of Type-II pnictogen bonding (Figure 13c,d). The tentative nature of intermolecular interactions inferred in this study between the O and Br sites of each molecule in the crystal is shown in Figure 13e. A similar topology of bonding is evident in crystalline POCl_3_ (ICSD ref. code 9128).

### 3.5. Diphosphorus Tetraiodide, P_2_I_4_

**Figure 14:**
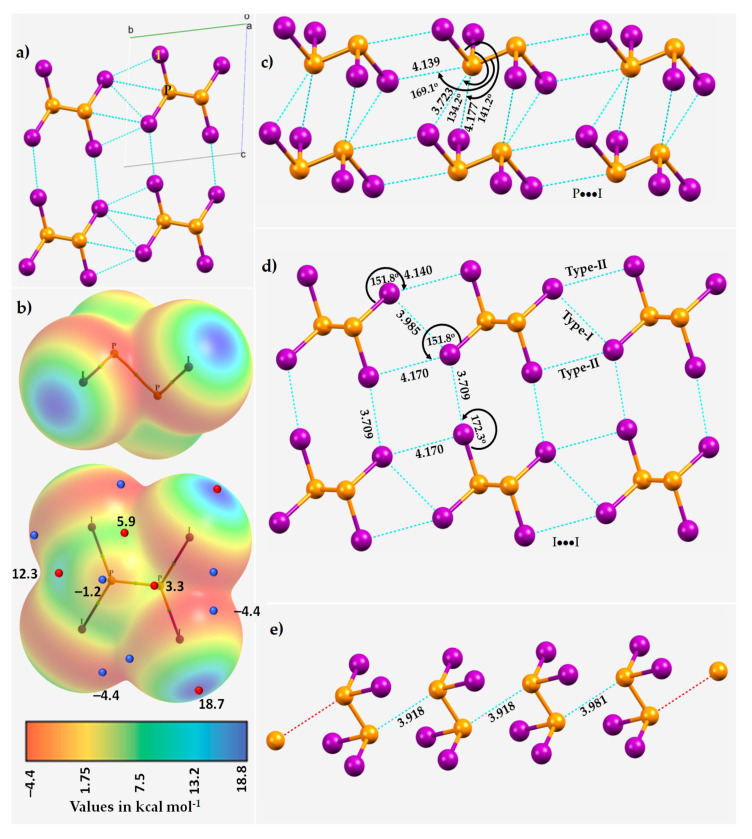
(**a**) The 2 × 2 × 2 supercell structure of the crystal structure of diphosphorus tetraiodide (ICSD ref. code 36293) [116] (space group P1). (**b**) The MP2(full)/def2-TZVPPD level 0.001 a.u. isodensity envelope mapped potential on the electrostatic surface of the P_2_I_4_ molecule, with selected local maxima and minima of potential marked by tiny red and blue spheres, respectively. (**c**–**e**) Illustration of the nature of P···I, I···I, and P···P intermolecular interactions in the crystal. Atom type is shown in (**a**). Selected bond distances and bond angles are in Å and degrees, respectively.

Diphosphorus tetraiodide (P_2_I_4_), ICSD ref. code 36293 [119], is an orange crystalline solid. The 2 × 2 supercell is shown in Figure 14a. The P_2_I_4_ units interact with each other through a number of intermolecular interactions. Before examining these interactions, we explored the nature of the potential on the electrostatic surface of a P_2_I_4_ molecule at the MP2(full)/def2-TZVPPD level of theory. The results, summarized in Figure 14b, suggest that the surface of I is most positive along the P–I bond extensions (*V_S,max_* = 18.7 kcal mol^−1^), and this potential maximum is surrounded by a belt of negative potential around the lateral sites (*V_S,min_* = –4.4 kcal mol^−1^). These results indicate I has sites that can act as a Lewis acid or a Lewis base when in proximity of another molecule in the crystalline state. Each of the two P atoms has a positive site along the P–P bond extension (*V_S,max_* = 12.3 kcal mol^−1^), and the expected lone-pair dominant region on P is lying perpendicular to the P–P bond axis (*V_S,min_* = –1.2 kcal mol^−1^).

As suggested by the MESP plot, we indeed observed that each P site in a P_2_I_4_ molecule makes Coulombic contact with the lateral negative sites on I in the surrounding molecules. As shown in Figure 14c, each P forms three P···I contacts that appear along the outer extensions of the P–P and I–P bonds. They are all different; one of them is shorter, and two are slighter longer than the sum of the vdW radii of P and I atoms, 3.94 Å (P (*r*_vdW_) = 1.90 Å; I (*r*_vdW_) = 2.04 Å). They are directional; one is quasilinear and two are nonlinear.

The Type-II I···I directional contacts locally form I_4_-type structures (Figure 14d). There are four such contacts that are σ-hole centered. Two of them have a bond distance of 3.709 Å, while the other two are substantially longer, with a distance of 4.170 Å. The first two are quasilinear while the last two are far off from linear. In addition, there are also Type-I and Type-II I···I contacts, similar to those shown in Figure 14d. In addition, we have also identified the possibility of P···P contacts (*r*(P···P) = 3.981 Å) between the P_2_I_4_ molecules in the crystal (Figure 14e). They are nonlinear and of π···π type. While these secondary interactions are the result of attractive engagements, their appearance must be influenced by the primary interactions in the crystal (viz. P···I and I···I). We note that the crystal structure of P_2_I_4_ has also been reported by others (ICSD ref. 426518 [120] and 203216 [121]); the noncovalent interactions in these structures are very comparable to those in the original structure [119] discussed above.

### 3.6. Miscellaneous Phosphorus Compounds

**Figure 15:**
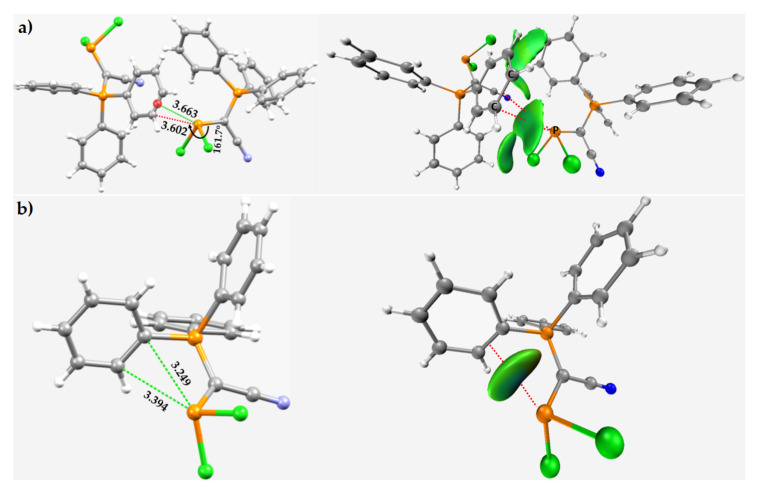
The P··· π(C=C) pnictogen bonding within the frameworks of the (**a**) dimer and (**b**) the monomer in the crystal structure of cyano(dichlorophosphanyl)(triphenyl-phosphaniumyl)-methanide, Ph_3_P^⊕^C^⊖^(PCl_2_)(CN) (CSD ref. NABRAN) [119]. The IGM-*δg*-based isosurface plots (isovalue = 0.005 a.u.) are shown in each case, displaying the possibility of intermolecular and intermolecular P···π(C=C) pnictogen bonds in (**a**) and (**b**), respectively. Selected bond distances and bond angles are shown in Å and degrees, respectively. The dotted line between atoms in green represents the inter/intramolecular interaction.

**Figure 16:**
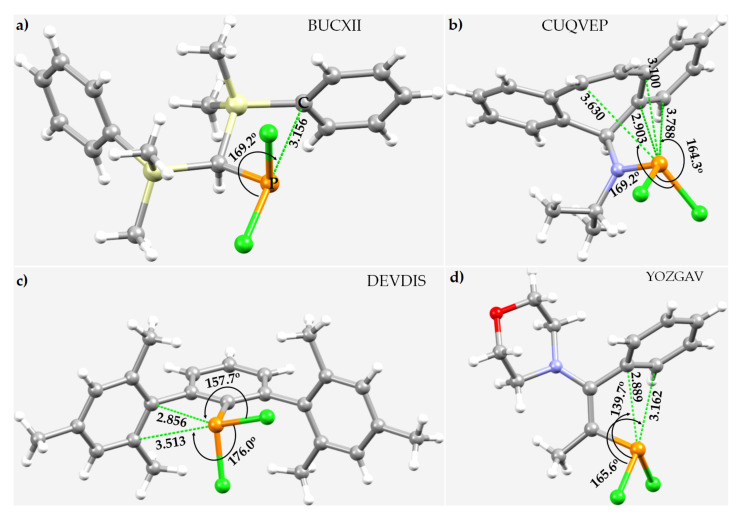
The P···π(C/C=C) intramolecular pnictogen bonding interactions in the crystals of (**a**) (bis(dimethyl(phenyl)silyl)methyl)(chloro)phosphine, ((CH_3_)_2_SiPh)_2_CH(PCl_2_) [120]; (**b**) *N*-(2,3:6,7-dibenzohepta-2,4,6-trienyl)-*N*-(dichlorophosphino)isopropylamine, RN(iPr)(PCl_2_) [121]; (**c**) dichloro-(2,6-bis(2,4,6-trimethylphenyl)phenyl)phosphane, RPCl_2_ [122]; and (**d**) 2-(dichlorophosphanyl)-1-morpholino-1-phenylpropene, 2-R-PCl_2_ [123]. Selected bond distances and angles are in Å and degrees, respectively. CSD reference is shown for each case. The dotted line in green represents the intramolecular interaction.

The examples of phosphorus-centered pnictogen bonding given above arose principally from an interaction between the positive site on the surface of P in molecule(s) and the lone-pair electron density on a halogen derivative, or oxygen, or nitrogen, etc., in the partner molecules. Shown below (Figure 15 and Figure 16) are a set of examples where the occurrence of phosphorus-centered pnictogen bonding occurs between the covalently bonded P and the π-density on a C atom, or the C=C double bonds, of arene moieties. The bonding may be intermolecular or intramolecular. There are many such chemical systems cataloged in the CSD, and we have chosen a few as examples to illustrate the occurrence of the interaction.

The dimeric arrangement between neighboring molecular entities in the crystal of Ph_3_P^⊕^C^⊖^(PCl_2_)(CN) [122] is shown in Figure 15a (left), while Figure 15b shows the monomeric unit. In the dimer, we observed a C–P···C_π_/π(C=C) pnictogen bond with an intermolecular bond distance of 3.602/3.663 Å. The presence of this interaction was confirmed by the IGM-*δg*-based isosurface plot shown as greenish flat volume in Figure 15a (right). The P atom is also bonded with the C=C double-bond arene within the skeletal framework of the monomer, thus forming an intramolecular C–P···C_π_/π(C=C) pnictogen bond (Figure 15b (right)), confirmed by the IGM-*δg* isosurface plot shown in Figure 15b (right). In both cases, the inter/intramolecular distance is less than the sum of the vdW radii of C and P atoms, 3.67 Å (*r_vdW_*(C) = 1.77 Å and *r_vdW_*(P) = 1.90 Å).

The molecular units in the crystals of ((CH_3_)_2_SiPh)_2_CH(PCl_2_) [123], RN(iPr)(PCl_2_) (R = 2,3:6,7-dibenzohepta-2,4,6-triene) [124], RPCl_2_ (R = 2,6-dimesitylphenyl) [125], and 2-R-PCl_2_ (R = 1-morpholino-1-phenylpropene) [126] are shown in Figure 16a–d. In all cases, C–P···C_π_/π(C=C) intramolecular pnictogen bonds play some role in the structural integrity of the crystalline material. They are directional and somewhat shorter than those observed in Ph_3_P^⊕^C^⊖^(PCl_2_)(CN) [122]. It must be appreciated, however, that there are numerous H···Cl and π···π, π···H interactions in the crystal, as shown in Figure 16b (right), that are at interplay between the molecular building blocks.

## 4. Conclusions

In this overview article, we have reexamined the geometric aspects of phosphorus-centered intra- and inter-molecular interactions in several crystal structures, selected for illustrative purposes, that have been deposited in the CSD and ICSD databases. We noted that the intermolecular distances of phosphorus bonds can be smaller, or larger, than the sum of the vdW radii of the respective atomic basins. In many cases, they are smaller, and this could be successfully explained as attractive interactions that occur between interacting regions of positive and negative potential, elucidated using the MESP model. While this overview article did not explore the details of other secondary (or even primary) interactions that appear simultaneously with pnictogen bonds, the former interactions either reinforce the stability of the crystal or enable the occurrence of phosphorus bonding. It was also observed that trivalent and higher-valent phosphorus atoms in molecules can feature three or more σ-holes along the R–P covalent bond extensions and that they, as phosphorus donors, have the ability to simultaneously engage attractively with several negative sites to form a variety of crystals cataloged in the crystal structure databases such as ICSD and CSD. We observed that the electron donors responsible for the formation of pnictogen bonding are not limited to the halogen derivative in the partner fragment/molecular entity but also include oxygen, nitrogen, carbon, phosphorus, and π-density in C=C double bonds or in a C≡N triple bond. We have demonstrated that in the case of molecular entities containing less polarizable atoms such as F, the proper choice of the isoelectronic density envelope is necessary on which to compute the electrostatic potential. We believe that the illustrative systems discussed in this overview will guide researchers in rationalizing phosphorus-centered pnictogen bonding in the rational design of molecular complexes, crystals, and materials and their subsequent characterization.

## Figures and Tables

**Table 1:** The 0.001 a.u. isodensity envelope mapped potential on the electrostatic surface of PX_3_ (X = F, Cl, Br, I) molecules, obtained using MP2(full)/def2-TZVPD ^a^.

Local Extrema on the Surface of Specific Atom/Bond	PF_3_	PF_3_	PCl_3_	PBr_3_	PI_3_
	0.001 a.u.	0.0028 a.u.	0.001 a.u.	0.001 a.u.	0.001 a.u.
*V_s,min_* On X (lateral portions)	−7.7	−7.5	−4.2	−4.4	−3.7
*V_s,min_* On X (lateral portions)	−7.7	−7.5	−4.2	−4.4	−3.7
*V_s,min_* On X (lateral portions)	−7.7	−7.5	−4.2	−4.4	−3.7
*V_s,min_* on P (opposite to the triangular face formed by three X atoms)	0.03	0.1	1.4	2.3	1.6
*V_S,max_* (on P−X bond extensions)	-	−5.3	10.5	14.4	19.1
*V_S,max_* (on P−X bond extensions)	-	−5.3	10.5	14.3	19.1
*V_S,max_* (on P−X bond extensions)	29.1	−5.3	10.5	14.4	19.1
*V_S,max_* (on X−P bond extensions)	29.1	48.9	20.7	18.5	14.9
*V_S,max_* (on X−P bond extensions)	29.1	48.9	20.8	18.5	14.8
*V_S,max_* (on X−P bond extensions)	9.5	48.9	20.8	18.4	14.8
*V_S,max_* (on the centroid of the triangular face formed by three X atoms)	-	26.0	3.0	2.3	2.4

^a^ The 0.0028 a.u. isodensity envelope mapped potential on the electrostatic surface of PF_3_ molecule is included to show that the σ-hole on F in this molecule is not neutral.

## Data Availability

This research did not report any data.
